# Alcohol in the Treatment of Disease

**Published:** 1896-10

**Authors:** 


### Alcohol in the Treatment of Disease.

*The Southwestern Medical and Surgical Reporter*, in a very interesting editorial, has this to say on the above subject:

"The time was when alcohol was believed by the majority of the medical profession to be the ideal stimulant in typhoid fever, pneumonia—particularly in the latter stages—and, in fact, all cases of anæmia or collapse or shock, or in any and all conditions of the system in which the vital powers were, by any means, brought below par.

"Men in the front rank in the profession held out, and a few are still holding out, that alcohol augmented the heart-force, and by that means increased arterial tension, and if given, when, for any reason, the heart was unable to properly fill the arteries, and especially those at a distance from the heart, the heart's energy would be revived and the results would be beneficial; and this all in the face of what physiologists had taught many years ago—that alcohol was rather a depressant of the vital powers than a stimulant.

"When we know the immediate effects of a large dose of alcohol upon the coats of the stomach, and when we stop to reflect that one of the most apparent effects of alcohol upon the system at large is a benumbing of the sensibilities, how can we doubt its depressing influence?

"Real stimulants do not act in this way, but their effect is to increase the tonicity of the nerve-supply throughout the system, by stimulating the heart and causing it to beat with more energy, thereby increasing sensibility and giving vigor to all the functions of the body.

"The concensus of opinion is now, we believe, that alcohol holds a very limited place in medical therapeutics, and should rarely be used—surely never as a stimulant."

